# Diethyl 2,6-dimethyl-4-(5-phenyl-1*H*-pyrazol-4-yl)-1,4-dihydro­pyridine-3,5-dicarboxyl­ate

**DOI:** 10.1107/S1600536812008173

**Published:** 2012-02-29

**Authors:** Hoong-Kun Fun, Chin Wei Ooi, Shridhar Malladi, K. N. Shivananda, Arun M. Isloor

**Affiliations:** a X-ray Crystallography Unit, School of Physics, Universiti Sains Malaysia, 11800 USM, Penang, Malaysia; b Medicinal Chemistry Division, Department of Chemistry, National Institute of Technology–Karnataka, Surathkal, Mangalore 575 025, India; c Schulich Faculty of Chemistry, Technion Israel Institute of Technology, Haifa 32000, Israel

## Abstract

In the title compound, C_22_H_25_N_3_O_4_, the dihydro­pyridine ring adopts a flattened boat conformation. The pyrazole ring makes a dihedral angle of 29.04 (5)° with the benzene ring. The mol­ecular structure is stabilized by an intra­molecular C—H⋯O hydrogen bond which generates an *S*(9) ring motif. In the crystal, mol­ecules are linked *via* N—H⋯O and C—H⋯N hydrogen bonds into a two-dimensional network parallel to the *ab* plane. The crystal structure is further consolidated by weak C—H⋯π inter­actions.

## Related literature

For details and applications of dihydro­pyridine, see: Stout & Meyers (1982 ▶); Böcker & Guengerich (1986 ▶); Vo *et al.* (1995 ▶). For hydrogen-bond motifs, see: Bernstein *et al.* (1995 ▶). For ring conformation, see: Cremer & Pople (1975 ▶). For a related structure, see: Fun *et al.* (2011 ▶). For bond-length data, see: Allen *et al.* (1987 ▶). For the stability of the temperature controller used for data collection, see: Cosier & Glazer (1986 ▶).
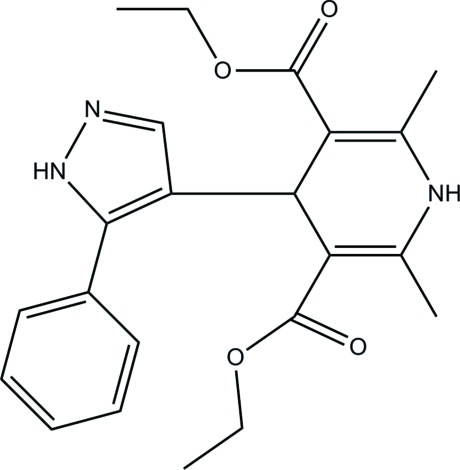


## Experimental

### 

#### Crystal data

C_22_H_25_N_3_O_4_
*M* *_r_* = 395.45
Monoclinic, 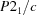

*a* = 9.7700 (4) Å
*b* = 8.6431 (4) Å
*c* = 24.8878 (9) Å
β = 105.646 (2)°
*V* = 2023.73 (14) Å^3^
*Z* = 4
Mo *K*α radiation
μ = 0.09 mm^−1^
*T* = 100 K
0.32 × 0.32 × 0.20 mm

#### Data collection

Bruker APEX DUO CCD area-detector diffractometer
Absorption correction: multi-scan (*SADABS*; Bruker, 2009 ▶) *T*_min_ = 0.972, *T*_max_ = 0.982
27563 measured reflections
7361 independent reflections
5987 reflections with *I* > 2σ(*I*)
*R*_int_ = 0.031

#### Refinement

*R*[*F* ^2^ > 2σ(*F* ^2^)] = 0.041
*wR*(*F* ^2^) = 0.118
*S* = 1.04
7361 reflections
266 parameters
H-atom parameters constrained
Δρ_max_ = 0.47 e Å^−3^
Δρ_min_ = −0.19 e Å^−3^

### 

Data collection: *APEX2* (Bruker, 2009 ▶); cell refinement: *SAINT* (Bruker, 2009 ▶); data reduction: *SAINT*; program(s) used to solve structure: *SHELXTL* (Sheldrick, 2008 ▶); program(s) used to refine structure: *SHELXTL*; molecular graphics: *SHELXTL*; software used to prepare material for publication: *SHELXTL* and *PLATON* (Spek, 2009 ▶).

## Supplementary Material

Crystal structure: contains datablock(s) global, I. DOI: 10.1107/S1600536812008173/is5081sup1.cif

Structure factors: contains datablock(s) I. DOI: 10.1107/S1600536812008173/is5081Isup2.hkl

Supplementary material file. DOI: 10.1107/S1600536812008173/is5081Isup3.cml

Additional supplementary materials:  crystallographic information; 3D view; checkCIF report

## Figures and Tables

**Table 1:** Hydrogen-bond geometry (Å, °) *Cg*1 and *Cg*2 are the centroids of the N1/N2/C7–C9 and C1–C6 rings.

*D*—H⋯*A*	*D*—H	H⋯*A*	*D*⋯*A*	*D*—H⋯*A*
N3—H1⋯O3^i^	0.89	2.09	2.9597 (12)	167
N1—H2⋯O1^ii^	0.90	1.96	2.8506 (10)	171
C3—H3*A*⋯N2^iii^	0.93	2.51	3.4202 (13)	164
C5—H5*A*⋯O4	0.93	2.50	3.4266 (12)	172
C21—H21*C*⋯N2^iv^	0.96	2.45	3.3300 (14)	153
C16—H16*A*⋯*Cg*1^v^	0.97	2.80	3.5318 (11)	133
C17—H17*C*⋯*Cg*2	0.96	2.99	3.7750 (15)	140
C19—H19*A*⋯*Cg*2^vi^	0.97	2.88	3.7079 (11)	144

Symmetry codes: (i) 
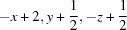
; (ii) 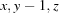; (iii) 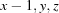; (iv) 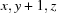; (v) 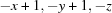; (vi) 
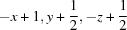
.

**Fig. 1.**
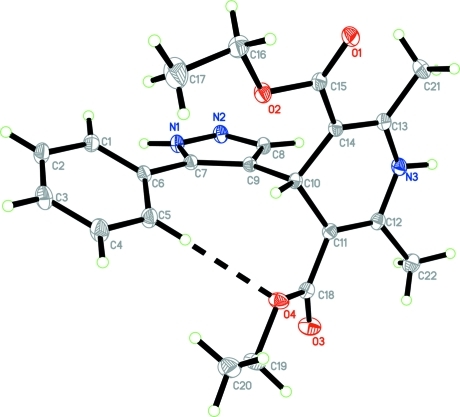
The molecular structure of the title compound, showing 50% probability displacement ellipsoids and the atom-numbering scheme. Intramolecular hydrogen bond was shown as dash line.

**Fig. 2.**
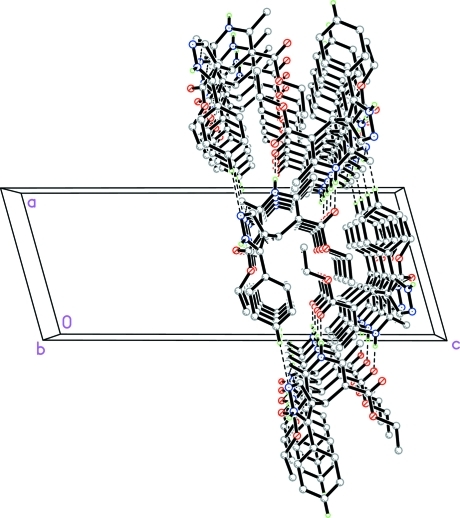
The crystal packing of the title compound, viewed along the b axis. H atoms not involved in the intermolecular interactions (dashed lines) have been omitted for clarity.

## supplementary crystallographic information

### Comment

1,4-Dihydropyridine (DHP) (Stout & Meyers, 1982) scaffold is a heterocyclic unit with remarkable pharmacological efficiency. They are widely used clinically as calcium channel blockers for the treatment of cardiovascular diseases. For example, nifedipine and nitrendipine are used for the treatment of hypertension and angina pectorism with nisoldipine being a potent vasodilator and nimodipine exhibiting selectivity for cerebral vasculature (Böcker & Guengerich, 1986). A number of DHP derivatives are employed as potential drug candidates for the treatment of congestive heart failure (Vo *et al.*, 1995). Prompted by the diverse activities of 1,4-dihydropyridines, we have synthesized the title compound to study its crystal structure.

In the title compound (Fig. 1), the dihydropyridine (N3/C10–C14) ring adopts a flattened boat conformation with puckering parameters (Cremer & Pople, 1975) Q = 0.2966 (9) Å, θ = 73.57 (17)° and φ = 185.61 (19)°. The pyrazole ring (N1/N2/C7–C9) is essentially planar [maximum deviation of 0.003 (1) Å at atoms C8 and C9] and makes a dihedral angle of 29.04 (5)° with the benzene ring (C1–C6). The molecular structure is stabilized by an intramolecular C5—H5A···O4 hydrogen bond (Table 1) which generates an S(9) ring motif (Bernstein *et al.*, 1995). The bond lengths (Allen *et al.*, 1987) and angles are within normal ranges are comparable to the related structure (Fun *et al.*, 2011).

In the crystal structure (Fig. 2), the molecules are linked *via* intermolecular N3—H1···O3, N1—H2···O1, C3—H3A···N2 and C21—H21C···N2 hydrogen bonds (Table 1) into two-dimensional networks parallel to the *ab* plane. The crystal structure is further consolidated by weak C—H···π interactions, involving the centroids of the pyrazole ring (N1/N2/C7–C9 *Cg*1; Table 1) and benzene ring (C1–C6 *Cg*2; Table 1).

### Experimental

3-Phenyl-1*H*-pyrazole-4-carbaldehyde (0.172 g, 1.0 mmol), ethylacetoacetate (0.26 g, 2.0 mmol) and ammonium acetate (0.092 g, 1.2 mmol) in ethanol (7 ml) were refluxed for 5 h. After the completion of the reaction, the reaction mixture was concentrated and poured into crushed ice. The precipitated product was filtered and washed with water. The resulting solid was recrystallized from ethanol: water mixture. Yield: 0.285 g, 72.15%. *M.p.*: 476–478 K.

### Refinement

Atoms H1 and H2 were located in a difference map and were fixed at their found positions with *U*_iso_(H) = 1.2 *U*_eq_(N) (N—H = 0.8870 and 0.9024 Å). The remaining H atoms were positioned geometrically and refined using a riding model, with *U*_iso_(H) = 1.2 or 1.5*U*_eq_(C) (C—H = 0.93–0.98 Å). A rotating group model was applied to the methyl groups. In the final refinement, the outliners (1 3 3), (-2 3 0) and (-2 3 10) were omitted.

### Crystal data

**Table:**

C_22_H_25_N_3_O_4_	*F*(000) = 840
*M**_r_* = 395.45	*D*_x_ = 1.298 Mg m^−^^3^
Monoclinic, *P*2_1_/*c*	Mo *K*α radiation, λ = 0.71073 Å
Hall symbol: -P 2ybc	Cell parameters from 8661 reflections
*a* = 9.7700 (4) Å	θ = 3.3–32.7°
*b* = 8.6431 (4) Å	µ = 0.09 mm^−^^1^
*c* = 24.8878 (9) Å	*T* = 100 K
β = 105.646 (2)°	Block, colourless
*V* = 2023.73 (14) Å^3^	0.32 × 0.32 × 0.20 mm
*Z* = 4	

### Data collection

**Table:**

Bruker APEX DUO CCD area-detector diffractometer	7361 independent reflections
Radiation source: fine-focus sealed tube	5987 reflections with *I* > 2σ(*I*)
Graphite monochromator	*R*_int_ = 0.031
φ and ω scans	θ_max_ = 32.7°, θ_min_ = 1.7°
Absorption correction: multi-scan (*SADABS*; Bruker, 2009)	*h* = −14→14
*T*_min_ = 0.972, *T*_max_ = 0.982	*k* = −13→12
27563 measured reflections	*l* = −37→37

### Refinement

**Table:**

Refinement on *F*^2^	Primary atom site location: structure-invariant direct methods
Least-squares matrix: full	Secondary atom site location: difference Fourier map
*R*[*F*^2^ > 2σ(*F*^2^)] = 0.041	Hydrogen site location: inferred from neighbouring sites
*wR*(*F*^2^) = 0.118	H-atom parameters constrained
*S* = 1.04	*w* = 1/[σ^2^(*F*_o_^2^) + (0.0611*P*)^2^ + 0.510*P*] where *P* = (*F*_o_^2^ + 2*F*_c_^2^)/3
7361 reflections	(Δ/σ)_max_ < 0.001
266 parameters	Δρ_max_ = 0.47 e Å^−^^3^
0 restraints	Δρ_min_ = −0.19 e Å^−^^3^

### Special details

**Table:**

Experimental. The crystal was placed in the cold stream of an Oxford Cryosystems Cobra open-flow nitrogen cryostat (Cosier & Glazer, 1986) operating at 100.0 (1) K.
Geometry. All e.s.d.'s (except the e.s.d. in the dihedral angle between two l.s. planes) are estimated using the full covariance matrix. The cell e.s.d.'s are taken into account individually in the estimation of e.s.d.'s in distances, angles and torsion angles; correlations between e.s.d.'s in cell parameters are only used when they are defined by crystal symmetry. An approximate (isotropic) treatment of cell e.s.d.'s is used for estimating e.s.d.'s involving l.s. planes.
Refinement. Refinement of *F*^2^ against ALL reflections. The weighted *R*-factor *wR* and goodness of fit *S* are based on *F*^2^, conventional *R*-factors *R* are based on *F*, with *F* set to zero for negative *F*^2^. The threshold expression of *F*^2^ > σ(*F*^2^) is used only for calculating *R*-factors(gt) *etc*. and is not relevant to the choice of reflections for refinement. *R*-factors based on *F*^2^ are statistically about twice as large as those based on *F*, and *R*- factors based on ALL data will be even larger.

### Fractional atomic coordinates and isotropic or equivalent isotropic displacement parameters (Å^2^)

**Table:**

	*x*	*y*	*z*	*U*_iso_*/*U*_eq_	
O1	0.58615 (8)	0.77001 (8)	0.04877 (3)	0.01973 (14)	
O2	0.46241 (7)	0.57230 (8)	0.07072 (3)	0.01770 (14)	
O3	0.81230 (7)	0.30340 (9)	0.29673 (3)	0.02111 (15)	
O4	0.59607 (7)	0.39878 (9)	0.25573 (3)	0.01732 (14)	
N1	0.64943 (8)	0.08372 (9)	0.08152 (3)	0.01486 (14)	
H2	0.6195	−0.0121	0.0695	0.018*	
N2	0.78581 (8)	0.12321 (10)	0.08546 (3)	0.01612 (15)	
N3	0.93751 (8)	0.59581 (10)	0.17656 (3)	0.01588 (15)	
H1	1.0205	0.6446	0.1854	0.019*	
C1	0.34348 (10)	0.08036 (11)	0.05798 (4)	0.01656 (16)	
H1A	0.3823	0.0415	0.0305	0.020*	
C2	0.20104 (10)	0.05325 (12)	0.05454 (4)	0.01944 (18)	
H2A	0.1454	−0.0036	0.0249	0.023*	
C3	0.14161 (10)	0.11085 (13)	0.09525 (4)	0.02181 (19)	
H3A	0.0463	0.0930	0.0930	0.026*	
C4	0.22583 (10)	0.19531 (13)	0.13937 (4)	0.02233 (19)	
H4A	0.1863	0.2343	0.1667	0.027*	
C5	0.36860 (10)	0.22241 (12)	0.14327 (4)	0.01838 (17)	
H5A	0.4239	0.2786	0.1732	0.022*	
C6	0.42916 (9)	0.16544 (10)	0.10232 (4)	0.01384 (15)	
C7	0.58005 (9)	0.18941 (10)	0.10518 (3)	0.01267 (15)	
C8	0.80244 (9)	0.25795 (11)	0.11222 (4)	0.01455 (15)	
H8A	0.8866	0.3143	0.1208	0.017*	
C9	0.67828 (9)	0.30610 (10)	0.12621 (3)	0.01221 (15)	
C10	0.66775 (8)	0.45725 (10)	0.15560 (3)	0.01229 (14)	
H10A	0.5715	0.4685	0.1599	0.015*	
C11	0.77430 (9)	0.45646 (10)	0.21308 (3)	0.01313 (15)	
C12	0.90530 (9)	0.51818 (11)	0.22054 (4)	0.01474 (16)	
C13	0.83443 (9)	0.64531 (10)	0.13003 (4)	0.01427 (15)	
C14	0.69904 (9)	0.59162 (10)	0.12121 (3)	0.01304 (15)	
C15	0.58227 (9)	0.65490 (10)	0.07696 (4)	0.01430 (15)	
C16	0.33872 (10)	0.62787 (13)	0.02848 (4)	0.02195 (19)	
H16A	0.3522	0.6144	−0.0084	0.026*	
H16B	0.3235	0.7369	0.0341	0.026*	
C17	0.21408 (12)	0.53514 (17)	0.03417 (6)	0.0380 (3)	
H17A	0.1310	0.5647	0.0054	0.057*	
H17B	0.1985	0.5542	0.0701	0.057*	
H17C	0.2327	0.4271	0.0306	0.057*	
C18	0.73457 (9)	0.37847 (11)	0.25931 (4)	0.01416 (15)	
C19	0.54490 (10)	0.33281 (13)	0.30055 (4)	0.02049 (18)	
H19A	0.6029	0.3677	0.3365	0.025*	
H19B	0.5482	0.2207	0.2995	0.025*	
C20	0.39406 (10)	0.38793 (14)	0.29101 (4)	0.0240 (2)	
H20A	0.3571	0.3525	0.3209	0.036*	
H20B	0.3369	0.3475	0.2563	0.036*	
H20C	0.3919	0.4989	0.2898	0.036*	
C21	0.88930 (10)	0.75484 (12)	0.09397 (4)	0.01890 (17)	
H21A	0.8378	0.7398	0.0556	0.028*	
H21B	0.9885	0.7354	0.0984	0.028*	
H21C	0.8767	0.8594	0.1048	0.028*	
C22	1.02486 (10)	0.51553 (13)	0.27302 (4)	0.02183 (19)	
H22A	0.9870	0.5049	0.3046	0.033*	
H22B	1.0778	0.6102	0.2761	0.033*	
H22C	1.0864	0.4297	0.2719	0.033*	

### Atomic displacement parameters (Å^2^)

**Table:**

	*U*^11^	*U*^22^	*U*^33^	*U*^12^	*U*^13^	*U*^23^
O1	0.0228 (3)	0.0136 (3)	0.0205 (3)	−0.0022 (3)	0.0019 (3)	0.0027 (2)
O2	0.0125 (3)	0.0169 (3)	0.0205 (3)	−0.0013 (2)	−0.0009 (2)	0.0040 (2)
O3	0.0191 (3)	0.0240 (4)	0.0201 (3)	0.0072 (3)	0.0052 (2)	0.0062 (3)
O4	0.0129 (3)	0.0244 (4)	0.0156 (3)	0.0005 (2)	0.0055 (2)	0.0038 (2)
N1	0.0135 (3)	0.0128 (3)	0.0189 (3)	−0.0013 (3)	0.0055 (3)	−0.0025 (3)
N2	0.0128 (3)	0.0155 (4)	0.0205 (3)	−0.0002 (3)	0.0053 (3)	−0.0011 (3)
N3	0.0111 (3)	0.0180 (4)	0.0180 (3)	−0.0029 (3)	0.0031 (3)	0.0005 (3)
C1	0.0158 (4)	0.0175 (4)	0.0166 (4)	−0.0030 (3)	0.0046 (3)	−0.0020 (3)
C2	0.0148 (4)	0.0208 (4)	0.0214 (4)	−0.0044 (3)	0.0027 (3)	−0.0024 (3)
C3	0.0139 (4)	0.0231 (5)	0.0293 (5)	−0.0038 (3)	0.0073 (3)	−0.0022 (4)
C4	0.0173 (4)	0.0260 (5)	0.0268 (5)	−0.0043 (4)	0.0112 (3)	−0.0063 (4)
C5	0.0155 (4)	0.0208 (4)	0.0202 (4)	−0.0044 (3)	0.0071 (3)	−0.0052 (3)
C6	0.0128 (3)	0.0135 (4)	0.0157 (3)	−0.0022 (3)	0.0046 (3)	0.0000 (3)
C7	0.0124 (3)	0.0126 (4)	0.0134 (3)	−0.0010 (3)	0.0041 (3)	−0.0007 (3)
C8	0.0117 (3)	0.0141 (4)	0.0176 (4)	−0.0001 (3)	0.0035 (3)	0.0000 (3)
C9	0.0112 (3)	0.0120 (4)	0.0131 (3)	−0.0006 (3)	0.0028 (3)	0.0001 (3)
C10	0.0109 (3)	0.0125 (4)	0.0132 (3)	−0.0003 (3)	0.0027 (3)	−0.0006 (3)
C11	0.0114 (3)	0.0145 (4)	0.0132 (3)	0.0002 (3)	0.0028 (3)	−0.0007 (3)
C12	0.0122 (3)	0.0157 (4)	0.0158 (4)	−0.0008 (3)	0.0028 (3)	−0.0020 (3)
C13	0.0140 (3)	0.0127 (4)	0.0163 (3)	−0.0007 (3)	0.0043 (3)	−0.0010 (3)
C14	0.0129 (3)	0.0115 (4)	0.0144 (3)	−0.0002 (3)	0.0031 (3)	−0.0004 (3)
C15	0.0147 (3)	0.0120 (4)	0.0157 (3)	−0.0006 (3)	0.0033 (3)	−0.0017 (3)
C16	0.0156 (4)	0.0219 (5)	0.0234 (4)	0.0011 (3)	−0.0033 (3)	0.0044 (4)
C17	0.0173 (5)	0.0401 (7)	0.0481 (7)	−0.0053 (5)	−0.0058 (5)	0.0133 (6)
C18	0.0129 (3)	0.0144 (4)	0.0150 (3)	0.0007 (3)	0.0035 (3)	−0.0015 (3)
C19	0.0205 (4)	0.0250 (5)	0.0185 (4)	−0.0006 (4)	0.0096 (3)	0.0046 (3)
C20	0.0181 (4)	0.0350 (6)	0.0214 (4)	−0.0031 (4)	0.0098 (3)	−0.0007 (4)
C21	0.0171 (4)	0.0170 (4)	0.0237 (4)	−0.0021 (3)	0.0074 (3)	0.0034 (3)
C22	0.0147 (4)	0.0285 (5)	0.0192 (4)	−0.0039 (4)	−0.0009 (3)	0.0004 (4)

### Geometric parameters (Å, º)

**Table:**

O1—C15	1.2239 (11)	C9—C10	1.5141 (12)
O2—C15	1.3442 (11)	C10—C14	1.5219 (12)
O2—C16	1.4529 (11)	C10—C11	1.5254 (11)
O3—C18	1.2181 (11)	C10—H10A	0.9800
O4—C18	1.3434 (10)	C11—C12	1.3524 (12)
O4—C19	1.4560 (11)	C11—C18	1.4730 (12)
N1—N2	1.3531 (10)	C12—C22	1.4993 (12)
N1—C7	1.3623 (11)	C13—C14	1.3626 (12)
N1—H2	0.9024	C13—C21	1.4995 (13)
N2—C8	1.3296 (12)	C14—C15	1.4613 (12)
N3—C13	1.3817 (11)	C16—C17	1.4961 (16)
N3—C12	1.3905 (11)	C16—H16A	0.9700
N3—H1	0.8870	C16—H16B	0.9700
C1—C2	1.3910 (12)	C17—H17A	0.9600
C1—C6	1.4002 (12)	C17—H17B	0.9600
C1—H1A	0.9300	C17—H17C	0.9600
C2—C3	1.3887 (14)	C19—C20	1.5055 (14)
C2—H2A	0.9300	C19—H19A	0.9700
C3—C4	1.3886 (14)	C19—H19B	0.9700
C3—H3A	0.9300	C20—H20A	0.9600
C4—C5	1.3918 (13)	C20—H20B	0.9600
C4—H4A	0.9300	C20—H20C	0.9600
C5—C6	1.3987 (12)	C21—H21A	0.9600
C5—H5A	0.9300	C21—H21B	0.9600
C6—C7	1.4713 (11)	C21—H21C	0.9600
C7—C9	1.3933 (12)	C22—H22A	0.9600
C8—C9	1.4120 (12)	C22—H22B	0.9600
C8—H8A	0.9300	C22—H22C	0.9600
			
C15—O2—C16	115.90 (7)	C14—C13—N3	119.09 (8)
C18—O4—C19	116.48 (7)	C14—C13—C21	127.35 (8)
N2—N1—C7	113.24 (7)	N3—C13—C21	113.56 (7)
N2—N1—H2	118.7	C13—C14—C15	121.38 (8)
C7—N1—H2	127.2	C13—C14—C10	120.18 (8)
C8—N2—N1	104.02 (7)	C15—C14—C10	118.36 (7)
C13—N3—C12	122.73 (7)	O1—C15—O2	121.75 (8)
C13—N3—H1	118.1	O1—C15—C14	126.70 (8)
C12—N3—H1	114.8	O2—C15—C14	111.53 (8)
C2—C1—C6	120.83 (8)	O2—C16—C17	107.09 (9)
C2—C1—H1A	119.6	O2—C16—H16A	110.3
C6—C1—H1A	119.6	C17—C16—H16A	110.3
C3—C2—C1	120.19 (9)	O2—C16—H16B	110.3
C3—C2—H2A	119.9	C17—C16—H16B	110.3
C1—C2—H2A	119.9	H16A—C16—H16B	108.6
C4—C3—C2	119.35 (8)	C16—C17—H17A	109.5
C4—C3—H3A	120.3	C16—C17—H17B	109.5
C2—C3—H3A	120.3	H17A—C17—H17B	109.5
C3—C4—C5	120.83 (9)	C16—C17—H17C	109.5
C3—C4—H4A	119.6	H17A—C17—H17C	109.5
C5—C4—H4A	119.6	H17B—C17—H17C	109.5
C4—C5—C6	120.19 (9)	O3—C18—O4	121.96 (8)
C4—C5—H5A	119.9	O3—C18—C11	126.86 (8)
C6—C5—H5A	119.9	O4—C18—C11	111.18 (7)
C5—C6—C1	118.60 (8)	O4—C19—C20	106.20 (8)
C5—C6—C7	122.03 (8)	O4—C19—H19A	110.5
C1—C6—C7	119.36 (8)	C20—C19—H19A	110.5
N1—C7—C9	105.97 (7)	O4—C19—H19B	110.5
N1—C7—C6	119.57 (8)	C20—C19—H19B	110.5
C9—C7—C6	134.41 (8)	H19A—C19—H19B	108.7
N2—C8—C9	112.62 (8)	C19—C20—H20A	109.5
N2—C8—H8A	123.7	C19—C20—H20B	109.5
C9—C8—H8A	123.7	H20A—C20—H20B	109.5
C7—C9—C8	104.15 (7)	C19—C20—H20C	109.5
C7—C9—C10	132.57 (7)	H20A—C20—H20C	109.5
C8—C9—C10	123.24 (7)	H20B—C20—H20C	109.5
C9—C10—C14	109.67 (7)	C13—C21—H21A	109.5
C9—C10—C11	109.33 (7)	C13—C21—H21B	109.5
C14—C10—C11	109.94 (7)	H21A—C21—H21B	109.5
C9—C10—H10A	109.3	C13—C21—H21C	109.5
C14—C10—H10A	109.3	H21A—C21—H21C	109.5
C11—C10—H10A	109.3	H21B—C21—H21C	109.5
C12—C11—C18	120.81 (8)	C12—C22—H22A	109.5
C12—C11—C10	120.55 (8)	C12—C22—H22B	109.5
C18—C11—C10	118.53 (7)	H22A—C22—H22B	109.5
C11—C12—N3	119.27 (8)	C12—C22—H22C	109.5
C11—C12—C22	126.85 (8)	H22A—C22—H22C	109.5
N3—C12—C22	113.87 (8)	H22B—C22—H22C	109.5
			
C7—N1—N2—C8	−0.22 (10)	C18—C11—C12—N3	−177.72 (8)
C6—C1—C2—C3	0.12 (15)	C10—C11—C12—N3	6.18 (13)
C1—C2—C3—C4	−0.14 (16)	C18—C11—C12—C22	1.13 (15)
C2—C3—C4—C5	−0.14 (17)	C10—C11—C12—C22	−174.97 (9)
C3—C4—C5—C6	0.44 (16)	C13—N3—C12—C11	15.77 (13)
C4—C5—C6—C1	−0.45 (15)	C13—N3—C12—C22	−163.22 (9)
C4—C5—C6—C7	−179.10 (9)	C12—N3—C13—C14	−13.03 (13)
C2—C1—C6—C5	0.17 (14)	C12—N3—C13—C21	167.59 (8)
C2—C1—C6—C7	178.86 (9)	N3—C13—C14—C15	171.93 (8)
N2—N1—C7—C9	−0.16 (10)	C21—C13—C14—C15	−8.78 (14)
N2—N1—C7—C6	177.68 (7)	N3—C13—C14—C10	−11.41 (13)
C5—C6—C7—N1	151.31 (9)	C21—C13—C14—C10	167.88 (8)
C1—C6—C7—N1	−27.33 (12)	C9—C10—C14—C13	−90.92 (9)
C5—C6—C7—C9	−31.60 (15)	C11—C10—C14—C13	29.33 (11)
C1—C6—C7—C9	149.76 (10)	C9—C10—C14—C15	85.84 (9)
N1—N2—C8—C9	0.52 (10)	C11—C10—C14—C15	−153.91 (7)
N1—C7—C9—C8	0.44 (9)	C16—O2—C15—O1	0.21 (13)
C6—C7—C9—C8	−176.93 (9)	C16—O2—C15—C14	178.67 (8)
N1—C7—C9—C10	178.03 (9)	C13—C14—C15—O1	−9.87 (14)
C6—C7—C9—C10	0.66 (17)	C10—C14—C15—O1	173.41 (9)
N2—C8—C9—C7	−0.62 (10)	C13—C14—C15—O2	171.76 (8)
N2—C8—C9—C10	−178.49 (8)	C10—C14—C15—O2	−4.96 (11)
C7—C9—C10—C14	−118.96 (10)	C15—O2—C16—C17	−171.85 (9)
C8—C9—C10—C14	58.24 (10)	C19—O4—C18—O3	2.98 (13)
C7—C9—C10—C11	120.42 (10)	C19—O4—C18—C11	−177.36 (8)
C8—C9—C10—C11	−62.38 (10)	C12—C11—C18—O3	−33.38 (14)
C9—C10—C11—C12	93.77 (10)	C10—C11—C18—O3	142.81 (9)
C14—C10—C11—C12	−26.69 (11)	C12—C11—C18—O4	146.98 (9)
C9—C10—C11—C18	−82.43 (9)	C10—C11—C18—O4	−36.83 (11)
C14—C10—C11—C18	157.11 (7)	C18—O4—C19—C20	172.95 (8)

### Hydrogen-bond geometry (Å, º)

*Cg*1 and *Cg*2 are the centroids of the N1/N2/C7–C9 and C1–C6 rings.

**Table:**

*D*—H···*A*	*D*—H	H···*A*	*D*···*A*	*D*—H···*A*
N3—H1···O3^i^	0.89	2.09	2.9597 (12)	167
N1—H2···O1^ii^	0.90	1.96	2.8506 (10)	171
C3—H3*A*···N2^iii^	0.93	2.51	3.4202 (13)	164
C5—H5*A*···O4	0.93	2.50	3.4266 (12)	172
C21—H21*C*···N2^iv^	0.96	2.45	3.3300 (14)	153
C16—H16*A*···*Cg*1^v^	0.97	2.80	3.5318 (11)	133
C17—H17*C*···*Cg*2	0.96	2.99	3.7750 (15)	140
C19—H19*A*···*Cg*2^vi^	0.97	2.88	3.7079 (11)	144

Symmetry codes: (i) −*x*+2, *y*+1/2, −*z*+1/2; (ii) *x*, *y*−1, *z*; (iii) *x*−1, *y*, *z*; (iv) *x*, *y*+1, *z*; (v) −*x*+1, −*y*+1, −*z*; (vi) −*x*+1, *y*+1/2, −*z*+1/2.
